# A bead-assisted flow cytometry method for the semi-quantitative analysis of Extracellular Vesicles

**DOI:** 10.1038/s41598-017-11249-2

**Published:** 2017-09-12

**Authors:** Henar Suárez, Ana Gámez-Valero, Raquel Reyes, Soraya López-Martín, María Josefa Rodríguez, José L. Carrascosa, Carlos Cabañas, Francesc E. Borràs, María Yáñez-Mó

**Affiliations:** 1 Instituto de Investigación Sanitaria La Princesa (IIS-IP), Madrid, Spain; 2 Departamento de Biología Molecular, UAM, Madrid, Spain; 3 REMAR-IVECAT Group, Health Science Research Institute Germans Trias i Pujol, Can Ruti Campus, Badalona, Spain; 4 Department of Pathology, Hospital Universitari and Health Sciences Research Institute Germans Trias i Pujol, Universitat Autònoma de Barcelona, Barcelona, Spain; 5 Centro de Biología Molecular Severo Ochoa, Madrid, Spain; 6 Departamento de Biología, Facultad de Ciencias, UAM, Madrid, Spain; 7 Department of Macromolecular Structures, Centro Nacional de Biotecnología-Consejo Superior de Investigaciones Científicas (CNB-CSIC), Madrid, Spain; 8 Nephrology Service, Germans Trias i Pujol University Hospital, Badalona, Spain

## Abstract

Most experimental approaches commonly employed for the characterization and quantitation of EVs are time consuming, require of specialized instrumentation and often are rather inaccurate. To circumvent the caveats imposed by EV small size, we used general and specific membrane markers in bead assisted flow cytometry, to provide a semi-quantitative measure of EV content in a given sample. EVs were isolated from *in vitro* cultured cells-conditioned medium and biological fluids by size exclusion chromatography and coupled to latex beads to allow their detection by standard flow cytometers. Our analyses demonstrate a linear correlation between EV concentration and Mean Fluorescence Intensity values in samples cleared of protein contaminants. Comparison with one of the most widespread method such as NTA, suggests a similar linear range and reliable accuracy to detect saturation. However, although detection of the different biomarkers is feasible when tested on ultracentrifugation-enriched samples, protein contamination impairs quantitation of this type of samples by bead-based flow cytometry. Thus, we provide evidence that bead-assisted flow cytometry method is an accurate and reliable method for the semiquantitative bulk analysis of EVs, which could be easily implemented in most laboratories.

## Introduction

Extracellular vesicles (EVs) include a variety of vesicles released to the extracellular media by most cell types as intercellular communication vehicles. EVs may transfer bioactive lipids, proteins, mRNA, miRNA or non-coding RNA, between cells^1, 2^. The term EV incorporates exosomes, microvesicles and apoptotic bodies, which differ in their size and origin. Exosomes have an endocytic origin and their diameter range between 30 and 150 nm. Microvesicles, however, originate by direct budding from the plasma membrane and are between 100 nm and 1 μm in diameter, while apoptotic bodies range from 1 μm to 5 μm and are released by dying cells^2^.

Besides their role in cell communication, EVs have recently emerged as a novel source of potential biomarkers for several diseases, since they can be easily obtained from body fluids such as urine^3, 4^, blood^5^, saliva or breast milk^4^ and their composition may be directly dependent on the physiological and/or pathological state of the patient. In addition, the number of EVs secreted can change upon the onset of different pathologies, so detecting variations in EV numbers could be of great relevance for diagnosis, especially in cancer patients^6^. Although there is a high heterogeneity in protein composition of EVs, yet, some proteins including tetraspanins, the *Tumour susceptibility gene 101* (Tsg101), Major Histocompatibility Complex molecules (MHC) or abundant GPI-linked molecules have been classically considered as common abundantly present elements on the surface of these vesicles^7–9^.

Because of their small size and heterogeneity, detection and quantitation of EVs have become difficult tasks. Some techniques have emerged, including Nanoparticle tracking analysis (NTA), that are currently the most widely employed –i.e. “the gold standard”- for their characterization. Its main restriction lies in the discrimination between vesicles and contaminating particles or protein aggregates present in the isolated sample, thus rendering inaccurate results in polydispersed samples.

To overcome some of these limitations, we propose a method allowing a semi-quantitative assessment of EVs in homogeneous and heterogeneous samples, reasonably unexpensive and easy to implement in any lab equipped with a standard flow cytometer. Our system is based on the detection of abundant EV proteins, such as tetraspanins, CD59 or MHC molecules by flow cytometry. To allow detection of EVs in conventional cytometers, vesicles are coupled to 4 μm diameter aldehyde-sulfate latex beads^10, 11^. Bead-based flow cytometry, usually coupling the EV-marker antibody to the beads, has been previously employed for EV characterization^12, 13^. Here we report a modification of that system for semi-quantitative analyses of EV samples. We directly use aldehyde-sulfate beads, not to exert any restriction in the binding process^14^ so that the total population of vesicles is included in the analysis. In this context, we demonstrate that linear changes in the MFI of several markers correlate with dilution of the samples thus allowing to build standard curves for the semi-quantitative evaluation of vesicles recovered from cell line cultures or biological fluids of interest. We provide evidence of the use of this method in EV isolated from tumor or primary cells as well as from human body fluids such as urine.

## Material and Methods

### Antibodies

Anti–HLA-A,B (clone W6/32;^15^), anti-CD59 (clone VJ1/ 12;^16^), anti-CD9 (clone VJ1/20) and anti-CD63 (clone TEA3/10), previously described^17^ and anti-CD81 (clone 5A6), kindly provided by S. Levy (Stanford University, CA), were used for the detection of EV-markers.

### Cells

SK-MEL103 human melanoma cell line was cultured in DMEM (GE Healthcare) supplemented with 10% Fetal bovine serum (FBS; GE Healthcare). Conditioned medium for EV isolation was collected twice a week. Primary T-lymphoblasts were isolated from human peripheral blood using a Ficoll (Biochrom) density gradient centrifugation as described elsewhere^18^. After activation with phytohaemagglutinin (PHA; Sigma) (1 µg/ml) for 24 h, 3 × 10^6^ cells/ml were cultured in RPMI (GE Healthcare) supplemented with 10% FBS and interleukin- 2 (IL2) (50 U/ml). Cells were diluted every two days to 3 × 10^6^ cells/ml in IL-2 containing media, and conditioned media was collected after one week of culture. FBS was depleted of bovine EVs by ultracentrifugation at 100,000xg for 16 h (Sorvall AH- 627 rotor, L8–70M ultracentrifuge, Beckman).

### EV isolation by size exclusion chromatography (SEC)

**Figure 1:**
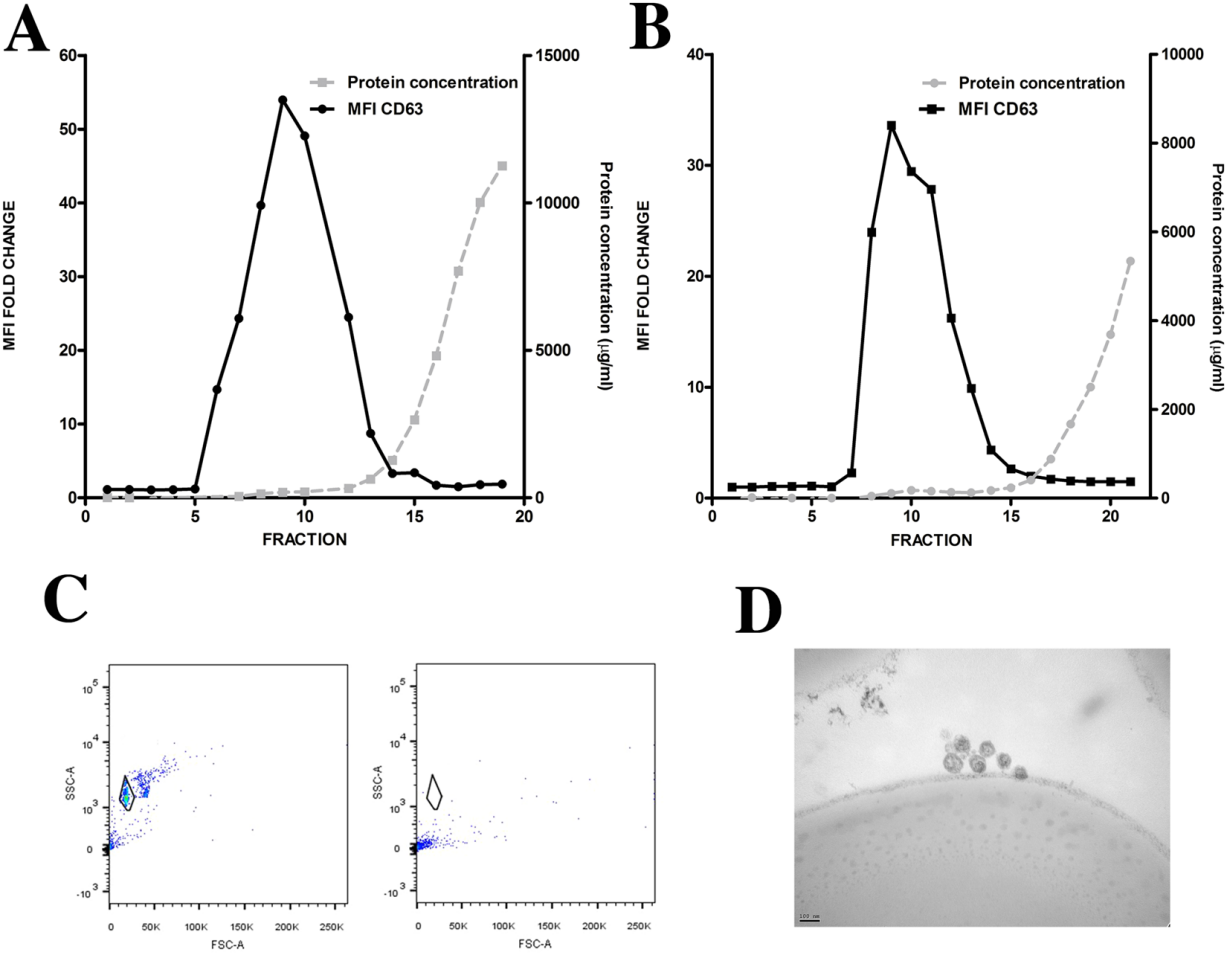
SEC-elution profile of EV samples. Conditioned media from melanoma cells (**A**) and T-lymphoblasts (**B**) cultures were processed using size exclusion chromatography (SEC). The obtained fractions were analysed by flow cytometry using anti-CD63 antibody after coupling EVs with aldehyde-sulfate beads. Protein concentration was also measured by BCA assay for each fraction and plotted in the right y-axis. Fold changes of mean fluorescence intensity (MFI) relative to the isotype control are plotted in the left y-axis. (**C**) Beads incubated with EVs (left) or EVs in solution (right) were incubated with primary and fluorochrome-labelled secondary antibodies. The flow cytometer gate used to analyse the beads is depicted in the SSC/FCS dot-plot. (**D**) Those beads showing CD63 positive staining were analysed by transmission electron microscopy. Bar: 100 nm.

Conditioned media or urine was recovered and centrifuged at 400 g for 5 minutes and at 2,000 g for 10 minutes to remove cells and cell debris. The cleared supernatant (45 mL) was concentrated by ultrafiltration at 2,000 g for 30 min using Amicon Ultra-15 Centrifugal Filter Units (Millipore, Billerica MA). This last step was repeated until the whole supernatant was concentrated to a final volume of 1.5 ml which was loaded onto a SEC column for EVs purification as previously described^19^. Briefly, Sepharose CL-2B (Sigma Aldrich) was stacked in a 20 ml syringe (BD, Plastipak), reaching a final matrix length of 4.3 cm and 2 cm in diameter and equilibrated with PBS. For EVs it has been reported that this setting will collect together particles larger than 70 nm^19^. Elution was performed by gravity with PBS, collecting 20 sequential fractions of 500 µl. Soluble protein elution was confirmed to be in the last fractions (15–20) using BCA assay. Detection of those fractions enriched in EVs was performed by bead-based flow cytometry analysis of each individual fraction using anti-CD63 antibody (Fig. 1). Only the three fractions with highest MFI values for this EV marker (commonly 8^th^–10^th^) were pooled for further EV downstream analyses.

### EV enrichment by Ultracentrifugation

Cell culture supernatants were recovered and centrifuged at 400 g for 5 minutes and at 2,000 g for 10 minutes to remove cells and cell debris. Supernatants were centrifuged again at 17,000 g for 20 minutes and then at 100,000 g for 2 h (Sorvall AH- 627 rotor, L8-70M ultracentrifuge, Beckman). The 100,000 g pellet was then washed with PBS (30 ml) and centrifuged again for 2 h at 100,000 g. EVs were resuspended in 1.5 ml of PBS.

### Flow cytometry assays

50 μl of each fraction isolated by SEC (diluted or not) or by ultracentrifugation were incubated with 0.25 μl of aldehyde/sulfate-latex beads (ø = 4 μm; 5.5 × 10^6^ particles/ml; Invitrogen, Carlsbad, CA) for 15 min at RT. Dilutions of the EV samples were performed with the same buffer used for their elution (PBS). Bead concentration was chosen to be large enough to be easily detected by the flow cytometer having enough events for proper statistics (over 5000), and small enough to use a small volume of sample. Then 1 ml of BCB (PBS supplemented with 0.1% BSA; Roche, and 0.01% NaN_3_; G-Biosciences) was added and the sample incubated overnight on rotation. Bead-coupled EVs were pelleted by centrifugation at 2,000 g for 10 minutes, washed with 1 ml of BCB and centrifuged again. The pellet was resuspended with 50 μl of BCB per analysis and stained using hybridoma supernatant of anti-MHC-I (W6/32), anti-CD59 (VJ1/12), anti-CD9 (VJ1/20), anti-CD63 (TEA3/10), anti-CD81(5A6) as primary antibodies and FITC-conjugated secondary antibodies (ThermoFisher Scientific) for 30 minutes at 4 °C. Negative control was obtained by incubating the beads coupled with the undiluted EV sample, with an isotype control followed by secondary antibody, or in the absence of primary mAb. Washing steps were performed once after primary and twice after secondary Ab labelling with 150 μl of BCB and centrifugation at 2,000 g for 10 minutes. Data was acquired in conventional flow cytometers (FacsVerse and FACSCanto A, BD Biosciences, San Jose, CA) and analysed with the FlowJo software (version Tree Star, Ashland, OR). Gating of EV-decorated 4 μm in diameter beads was performed based on FCS/SSC parameters, so that unbound EVs or possible antibody aggregates are excluded from the analysis.

### Protein concentration measurement

Pierce BCA Protein assay kit was used following manufacturer instructions. Detection provided by manufacturer, ranges between 5–2000 µg/ml.

### Nanoparticle tracking analysis

Size distribution and concentration of EVs was determined using NANOSIGHT LM10 (Malvern Instruments Ltd, Malvern, UK) equipped with charge-coupled device (CCD) camera (model F-033) and a 638 nm laser. Analysis was performed using the NTA 3.0 software. Detection threshold was set to 5, and blur and Max Jump Distance were set automatically. Diluted (100- to 1200-fold) fractions were loaded in the NTA device and 60 s videos were recorded in triplicate with the camera shutter at 30.02 ms and the camera gain at 650, as recommended by the manufacturer.

### Sample processing for Transmission Electron Microscopy (TEM)

For ultrastructural studies, a latex beads pellet was treated with a mixture of 2% formaldehyde (Ultra Pure EM Grade, Polysciences Inc., Philadelphia, USA) and 2.5% glutaraldehyde (EM Grade, TAAB Laboratories Equipment Ltd., Berks, UK) in PBS for 1 h at room temperature. The pellet was then washed with PBS and distilled water, post-fixed for 45 minutes with 1% osmium tetroxide (TAAB Laboratories Equipment Ltd.) in PBS, washed with distilled water, treated during 45 minutes with 1% aqueous uranyl acetate (Electron Microscopy Sciences, Hatfield, USA), washed again and dehydrated with growing quantities (30%, 50%, 70%, 90% and 100%) of ethanol seccosolv (Merck KGaA, Darmstadt, Germany) at room temperature. The sample was maintained in an eppendorf throughout the process and finally embedded in epoxy resin 812 (TAAB Laboratories Equipment Ltd.) contained in beem embedding capsule (TAAB, polythene truncated pyramid 5, 2 mm diameter). The epoxy resin was polymerized for 2 days at 60 °C after a 5 minutes-centrifugation step at 14500 rpm. Due to the properties of the latex beads, the sample remained in the opposite side of the truncated pyramid of the beem capsule. Ultrathin, 70-nm-thick sections were obtained from that side with an Ultracut UCT ultramicrotome (Leica Microsystems), transferred to buttonhole Nickel EM grids (GS2 × 1-N3, Gilder, Lincolnshire, UK) and stained with 3% aqueous uranyl acetate (10 minutes) and lead citrate (2 minutes) (Electron Microscopy Science). Sections were visualized on a JEOL JEM 1200 EXII electron microscope operating at 100 kV (JEOL Ltd., Tokyo, Japan).

### Statistical analysis

Statistical analyses and linear regressions were calculated with GraphPad Prism (GraphPad Software Inc).

Ethics statement: Experimental protocols for human samples were carried out following international regulations, including written consent by the donors, and approved by the Ethical Committee of the Hospital Universitario de la Princesa.

## Results

### Bead-assisted flow cytometry can be used as a highly sensitive semi-quantitative method for EV analysis

EVs were isolated from culture supernatants of human primary T-lymphoblasts or SK-MEL103 melanoma cells, using Size Exclusion Chromatography (SEC) to remove most protein contaminants from EV samples. All 20 fractions collected were analysed for protein content by BCA assay and characterized by bead-assisted flow cytometry using antibodies against classical EV-marker CD63 (Fig. 1A,B). Bead-assisted flow cytometry was performed by incubating the samples with 4 μm in diameter aldehyde/sulfate-latex beads. This size of the beads is such that they can be clearly detected and resolved from background signal by regular cytometers (Fig. 1C). No signal could be observed in the cytometric bead gate after incubating the EV samples with antibodies (primary and secondary), suggesting that even antibody-induced aggregates cannot be resolved from background in this kind of equipment (Fig. 1C). Electron microscopy analyses of beads incubated with the CD63 positive fractions demonstrated the presence of EV adsorbed on the surface of the beads (Fig. 1D). As previously described^10^, EVs detected as the CD63 positive fractions, were recovered before the elution of soluble proteins (Fig. 1A,B). Already from this initial analysis, it became apparent that the power of detection of bead-assisted flow cytometry is much higher than that of protein analyses such as the BCA assay (with detection range between 5–2000 µg/ml), which for vesicular fractions lay close or even below its detection limit.

**Figure 2:**
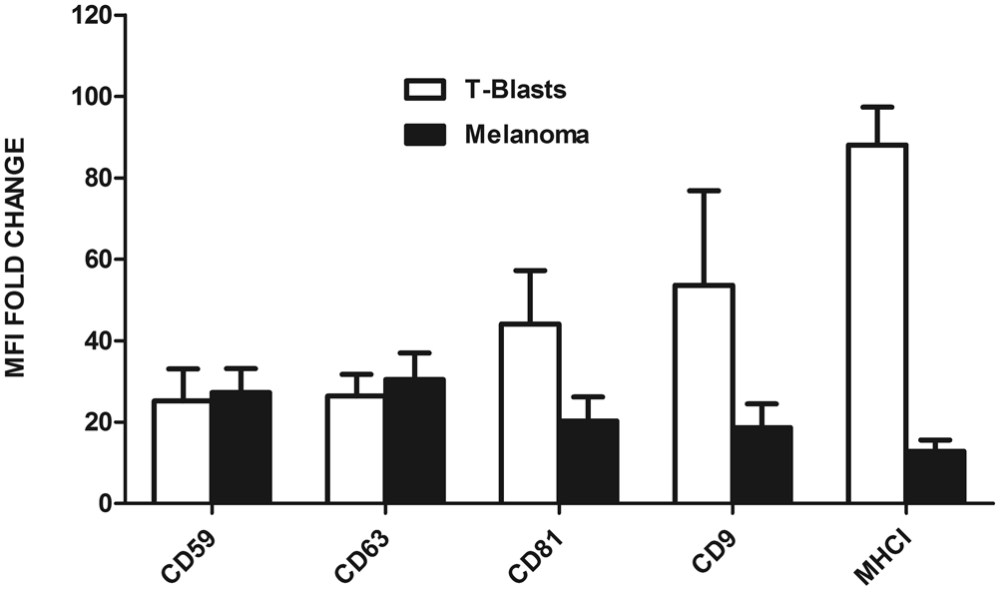
Characterization of EVs. T-lymphoblasts and melanoma EVs, isolated by SEC, were analysed by bead-based flow cytometry using antibodies against common EV markers. Antibodies used were anti-CD59 VJ1/12, anti-CD63 TEA3/10, anti-CD81 5A6, anti-CD9 VJ1/20, and anti-MHC-I W6/32 mAbs. Data correspond to the mean ± SEM of the MFI fold change referred to the negative control, in 3–5 independent experiments.

Given the high heterogeneity of EVs, we first assessed the most suitable markers for each EV sample, to be used in bead-assisted flow cytometry. The relative expression of EV markers in vesicles derived from two different cell cultures, SK-MEL103 melanoma cell line and primary human T-lymphoblasts, was assessed. The analysis included three different tetraspanins commonly employed as EV markers: CD9, CD81 and CD63. In addition, we assessed the expression levels of MHC-I molecules as well as the GPI-linked complement regulatory molecule CD59, which is highly expressed in most cell types^20^ (Fig. 2). Despite being these markers conserved in samples from different origins, expression levels can differ on the plasma membrane of the producing cells as well as on EVs. In our case, these differences were remarkable for CD81, CD9 and MHC class I (Fig. 2). This preliminary profiling has to be performed in order to select the appropriate combination of markers to measure the EVs produced by a given cell line or present in a specific biological fluid.

**Figure 3:**
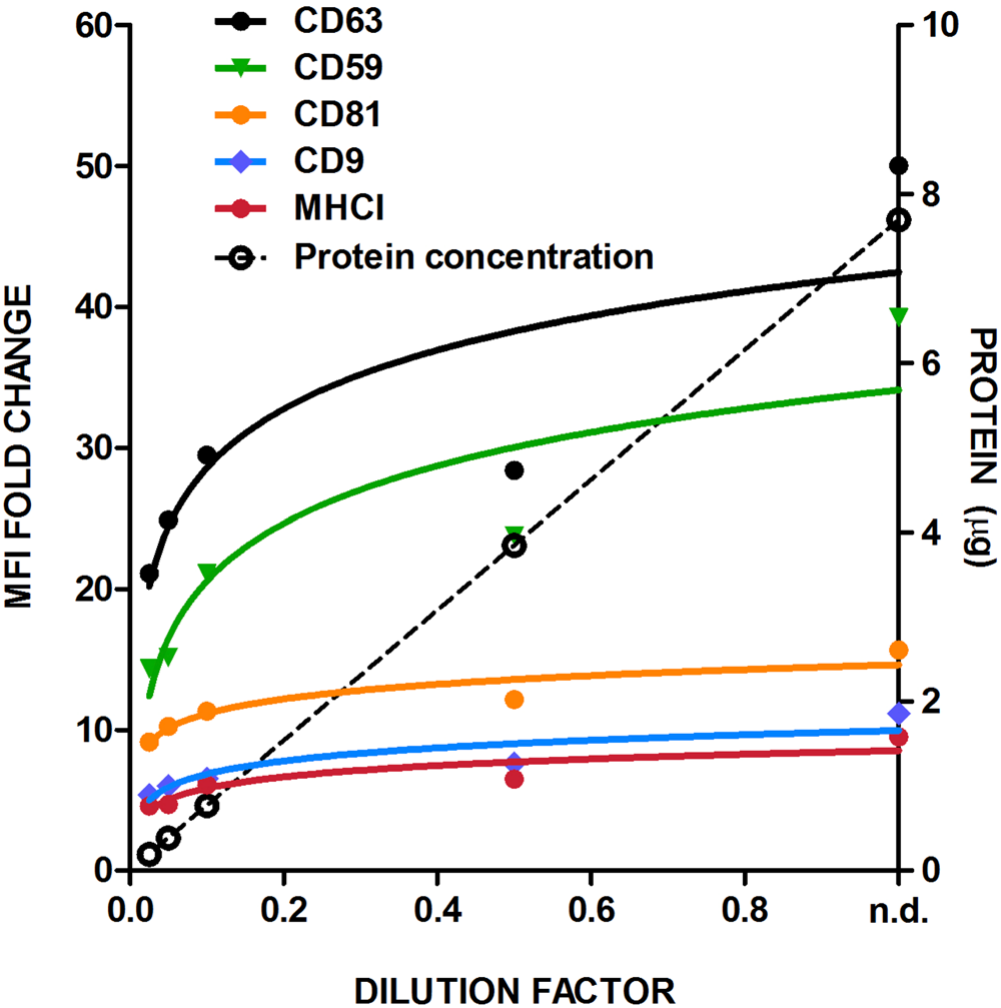
Analysis of antibody-staining detection of EV surface molecules in flow cytometry assays. Pooled SEC fractions containing melanoma EVs were sequentially diluted. Every dilution was analysed using a panel of antibodies: anti-CD63 (TEA3/10) in red, anti-CD59 (VJ1/12) in green, anti-CD81 (5A6) in yellow, anti-CD9 (VJ1/20) in purple and anti-MHCI (W6/32) in blue. The ratio of the MFI obtained relative to the negative control is plotted in the left y-axis. Protein content is plotted in the right y-axis and dotted line. The dilution factor of each measure is plotted on the x-axis (n.d.: non-diluted).

To assess whether the output of our bead-assisted flow cytometry method is in linear relation with EV concentration, we performed a set of EVs dilutions using melanoma EVs. Each individual EV dilution was coupled to the beads, incubated with the different antibodies and finally analysed by flow cytometry (Fig. 3). In the same samples, the protein content of each dilution was determined. As shown in Fig. 3, protein measurements exhibit a linear correlation in all the samples analysed, yet at higher EV dilutions (i.e. at lower dilution factor values) the values were close to the detection limit. In contrast, the MFI values for CD63 and CD59 detection still showed a linear response with these very diluted samples, while reflected saturation in less diluted samples (Fig. 3). In these particular melanoma vesicles, the expression levels of tetraspanins CD81 and CD9 as well as of MHC-I are limited, so that their changes in MFI values are small throughout the range of sample dilutions. These data strongly suggest that a high MFI is crucial for a good dynamic range and sensitivity of the quantitation method, as is the case for anti-CD63 and anti-CD59 in melanoma-derived EVs. In the case of T-lymphoblast-derived EVs, both CD63 and MHC-I were selected among those showing higher MFI to be used in the subsequent analyses.

**Figure 4:**
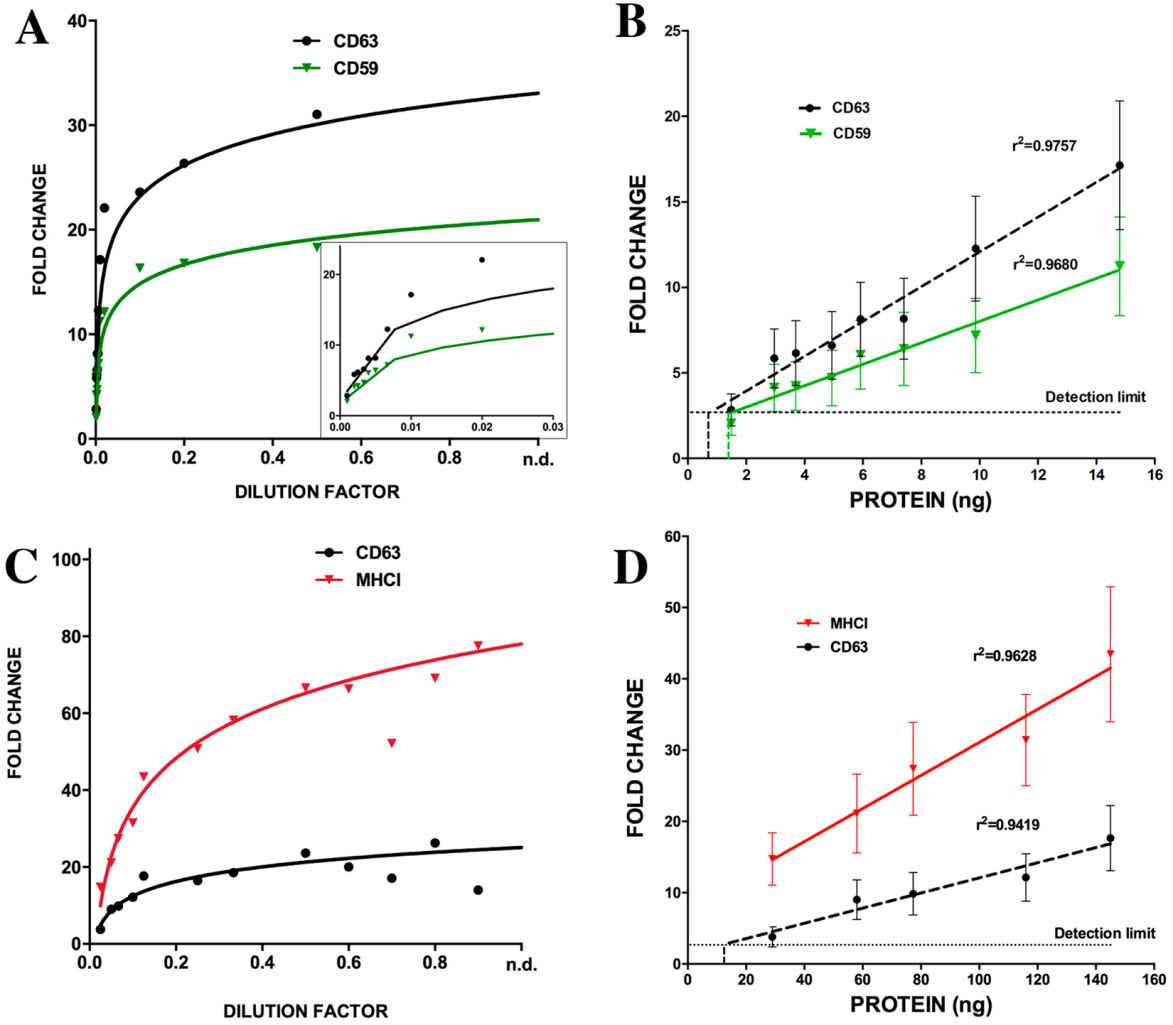
MFI values linearly correlate with EV content. EVs fractions were subjected to serial dilutions. Diluted EVs were coupled to beads and analysed by flow cytometry. Graphs on the top correspond to the data for melanoma-derived EVs and those on the bottom to data for T-lymphoblast-EVs. (**A**) and (**C**) EV dilution is plotted on the x-axis and fold change of MFI relative to negative control on the y-axis. Inset in A corresponds to the highest dilutions of the same graph. (**B**) and (**D**) Protein content of the undiluted fraction was measured using a BCA assay to represent MFI fold change (error bars correspond to SD values) versus protein content in the linear range (dilutions 1/100-1/1000 for melanoma-EVs and 1/8-1/40 for the T-lymphoblast-EV sample). Antibodies used were anti-CD63 TEA3/10 mAb, anti-CD59 VJ1/12 mAb and anti-MHC-I W6/32 mAb. The limit of detection of the measure, calculated as the background signal plus 3 times its SD, is shown as a horizontal dashed line. The detection limit with each marker is depicted with the corresponding colour as a vertical dashed line. The values for r^2^ are depicted in the graphs. In all cases p < 0.0001.

To estimate the range of linear detection as well as the detection limit of our measurement, we increased the range of dilutions of SEC-isolated EV samples analysed (Fig. 4). As for previous experiment, serial dilutions were coupled to beads and stained with anti-CD63 and anti-CD59 for melanoma-EVs or anti-CD63 and anti-MHC-I for T-lymphoblasts-EVs. MFI values were normalised relative to the negative control (isotype Ab-stained beads), and plotted against the dilution factor. A typical curve showing a saturated phase was observed when plotting the MFI fold-increase against the EV sample dilution (Fig. 4A,C). The protein content of the undiluted samples was determined by BCA to be 116.85 µg/ml in the case of T-lymphoblasts-EVs and 148.16 µg/ml for melanoma-EVs. We used these values to convert the linear range of the plot to MFI versus protein content (Fig. 4B,D). These analyses revealed a good linear behaviour, with very high r^2^ values and p values < 0.001, in the range between 1 to 15 ng for melanoma-EVs using anti-CD63 and anti-CD59. In the case of T-lymphoblasts-EVs, there was a good linear response in the range between 30 and 150 ng for MHCI and CD63 labelling. The limit of detection of the measure, usually determined as the background signal +3 times the standard deviation of the background signal, has been plotted in the figure, to determine the limit of EV detection in our assay. As shown in Fig. 4B,D, the limit of EV detection depends on the antibody used for staining, since it corresponds to the crossing point of the regression curve with the detection limit of the measure, but in all cases lay in the range of ng of vesicular proteins. The detection limit for MHCI in T-lymphoblast is really low, suggesting that we can indeed detect very few EVs with this marker. Larger dilutions should be analysed to have an accurate regression curve in this very diluted sample range, in order to obtain the exact limit of detection with this marker.

### Comparison of bead-assisted flow cytometry with Nanoparticle Tracking Analysis

**Figure 5:**
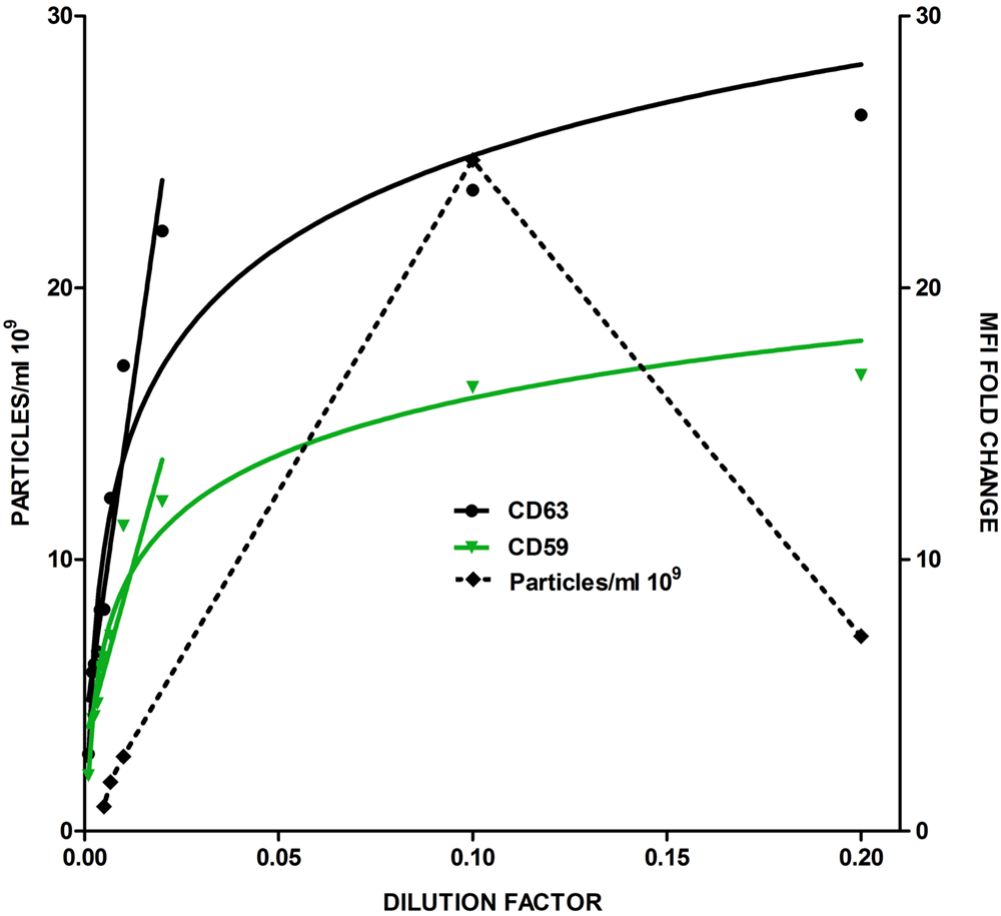
Comparison of flow cytometry-based and NTA quantitative analyses. A set of serial dilutions of melanoma-derived EVs were analysed NTA (particle concentration plotted on the left y-axis) and by bead-based assay (MFI folds of negative control plotted on the right y-axis as measured with anti-CD63 (TEA3/10) and anti-CD59 (VJ1/12) mAbs). The linear range of the fold change is also represented in the graph.

We next performed parallel analyses of different sample dilutions by the current “gold-standard” method in EV quantitation, NTA, and bead-assisted flow cytometry. As it can be seen in Fig. 5, the linearity of NTA measurements was clearly maintained for melanoma EV samples diluted from 10 to 200 times, that corresponded to 25–0.9 × 10^9^ vesicles, respectively. However, at higher concentrations (0.20 dilution factor), the result provided by NTA was smaller than the 10-times dilution, with no clear indication of saturating conditions. In comparison, the linear range of bead-assisted flow cytometer was narrower (in this case ranging from 50 to 200-times dilution (0.02–0.005 dilution factor), as depicted in Fig. 5). However, saturation of the measure occurred always at the same values of MFI for each marker (~15 folds the MFI value of the negative control for anti-CD63, ~8 folds for anti-CD59). If we calculate the number of EVs detected by the flow cytometry method, based on NTA absolute numbers, our detection range corresponds to 5 × 10^2^ to 3.5 × 10^3^ EVs/bead, suggesting that saturation of the flow cytometry signal occurs at the point were no more vesicles can be bound to beads, so the signal is maximum. This saturation point is therefore constant for each marker and sample type since when beads are completely covered by vesicles, the signal cannot increase anymore, independently on the antibody used for staining. In addition, sensitivity of this method, taken as the change in signal with changes in concentration, is higher than for NTA, since in the detection range, the slopes of the regression curves are very pronounced, suggesting that small changes in EV content are reflected in big changes in MFI.

Thus, for an unknown sample, our method enables its semi-quantitative analysis by measuring the fluorescence signal of the sample, while the parameters of the standard curve will give the operator information on the suited range of detection, based on the maximum MFI values reached for a given marker.

### Bead-assisted flow cytometry can be employed in biofluid-derived samples

**Figure 6:**
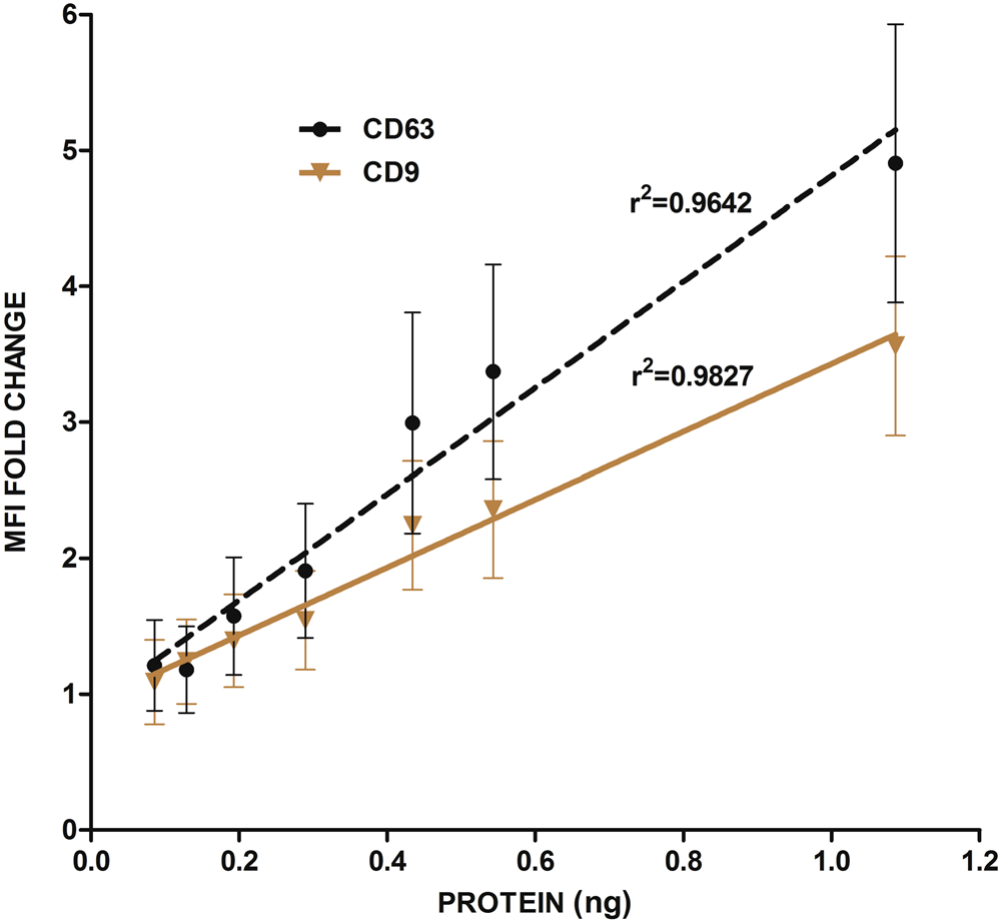
MFI values obtained in human urine samples also correlate with dilution factor and protein concentration. EVs isolated from urine by SEC, were submitted to sequential dilutions and analysed by flow cytometry using two antibodies: anti-CD63 (TEA3/10) in black and anti-CD9 (VJ1/20) in brown. Protein content of the undiluted SEC sample was measured by BCA assay. SD values and the values for r^2^ are depicted in the graphs. In all cases p <0.0001.

Since exosomes are becoming important biomarkers for many pathologies, we were interested in testing if this relation between MFI and EVs number/concentration, could be also applied to biological fluids. EVs from human urine where isolated and purified by SEC, and thereafter submitted to sequential dilutions to be measured by flow cytometry using antibodies against tetraspanins CD63 and CD9 (Fig. 6). As shown in the plot, EVs from human fluids also maintain a strong linearity between the amount of total protein and MFI values, as reflected by the good correlation parameters for both markers.

### The use of bead-assisted flow cytometry in protein-containing samples

**Figure 7:**
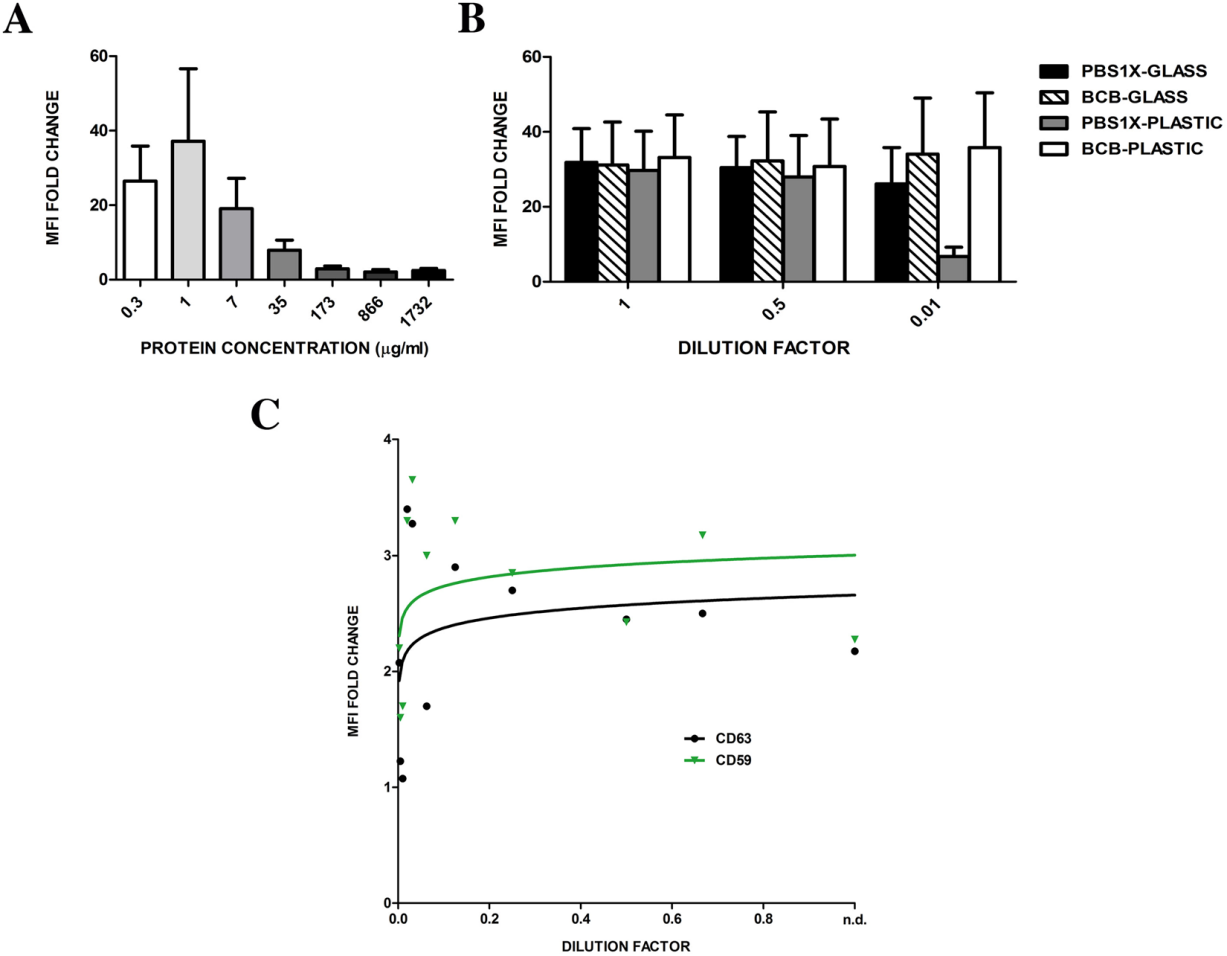
MFI determination is impaired by high levels of protein contamination. (**A**) EVs isolated by SEC from melanoma conditioned medium, were coupled to the beads in the presence of increasing amounts of protein from the last fractions of the SEC chromatography and analysed by flow cytometry using anti-CD63 (TEA3/10) antibody. Data correspond to the mean ± SEM of the MFI referred to that of the negative control, in three independent experiments. (**B**) Protein contamination greatly impairs the coupling to the beads, especially when performed in plastic tubes. Data correspond to the mean ± SEM of the MFI referred to that of the negative control, in three independent experiments. (**C**) Melanoma-EVs were concentrated by ultracentrifugation and subjected to serial dilutions before coupling to beads to be analysed by flow cytometry after staining with either anti-CD63 (TEA3/10) and anti-CD59 (VJ1/12) mAbs.

SEC has the clear advantage of greatly reducing protein contaminants in EV samples. However, the majority of the research in the EV field is still using serial ultracentrifugation to enrich EVs from cell culture supernatant or biofluids. To assess whether the presence of protein contaminants could interfere with our bead-assisted flow cytometry assay, we coupled SEC-isolated EVs with beads in the presence of increasing amounts of “contaminant protein”. To mimic as much as possible the composition of the original fluid, this “contaminant protein” was obtained from the protein-containing fractions after SEC purification of the same samples (fractions from 18 to 20). As observed in Fig. 7A, the presence of a small amount of protein (around 1 µg/ml) during the coupling process did not impair the detection of the EV marker by flow cytometry. A good signal could still be observed with protein contamination as high as 7 µg/ml, but the MFI values decreased thereafter.

The presence of some protein in the coupling buffer seems to favour the coupling of EVs to the heavily charged beads or, in addition, could prevent the adherence of EVs to the plastic tube, thus we next performed the coupling either in glass or plastic tubes and in the absence of protein or in our standard conditions (0.1% BSA (BCB)). These specific experiments were performed using highly concentrated samples, in the saturation phase of the curve, in which differences in MFI should only be due to effects on the coupling efficiency. As shown in Fig. 7B, in the presence of 0.1% BSA the coupling efficiency was equal for all samples. In the absence of BSA, however, a drastic decrease in MFI could be observed in the 10-times dilution when using plastic tubes. Thus, the presence of a small amount of protein either improves or does not affect detection of EVs, being this positive effect more evident in samples with scarcer EVs. In addition, these results suggest that some plastic tubes may be highly adhesive for EVs avoiding their coupling to the beads.

Finally, to assess the suitability of bead-assisted flow cytometry in ultracentrifuged samples, we performed similar analyses with melanoma EV samples enriched by ultracentrifugation (UC). Serial dilutions of the UC-pellet were performed before coupling to the beads. In sharp contrast to the previous results, in this case vesicular markers could be detected at very low signal intensity in most samples, and the linearity with concentration could not be observed (Fig. 7C).

## Discussion

Extracellular vesicle isolation and accurate quantification still remain as one of the main challenges in this field of research. The diverse isolation procedures described render samples with different levels of contaminants that may turn out to yield very disparate results. Lately, serial ultracentrifugation, the most commonly used EV-isolation method, is being questioned because of its lack of specificity, since it does not allow establishing a strict restriction over the presence of soluble proteins or aggregates. In this situation, SEC has emerged as an alternative advantageous method that overcomes many of the limitations of ultracentrifugation^21^, providing samples with high levels of intact vesicles and with reduced protein contamination by just appropriate selection of the pore size of the matrix.

Protein quantification has been commonly used as a fast method to determine the amount of vesicles present in a sample. However, these results have to be considered carefully; a direct correlation between the protein content and the number of vesicles in a given sample cannot be directly established, not only because of the highly heterogeneous nature of EVs, both in their size and composition, but mostly because of the high variability in the relative amount of accompanying protein contaminants co-enriched in the different isolation methods. These protein contaminants will cause an overestimation in the number of vesicles based not only on protein quantitation, but also on NTA analyses, since this latter technique cannot properly discriminate between EVs and large protein or contaminant aggregates. While SEC-based isolation may not produce completely pure EV samples^22^, it has the advantage of highly reducing protein contamination, thus permitting a more accurate and comparable quantitation of EVs.

The development of a reliable method to quantify EVs using flow cytometry requires using antibodies with a high sensitivity and a good linear response. Linearity involves a strong correlation between EV concentration and fluorescence signal. Mean Fluorescence Intensity (MFI) values depend on both the sample properties as well as on the combination of antibodies (primary/secondary) used for detection. As EVs size is under the resolution limit of any conventional cytometer, their coupling to latex beads becomes essential for their analysis using this system^12, 13^. There are now several commercially available kits of beads coupled to antibodies specific for EV markers, being CD63 the most widely used^23^. However, because of the high heterogeneity of these vesicles, it is especially important to ensure that there is no preference for any subpopulation of vesicles for their quantitative assessment. In this respect, we should remark that the proposed method is based on the bulk detection of EVs. Since a given bead will accommodate several EVs potentially expressing different sets of markers, in the end, each fluorescent dot detected in the flow cytometer suppose a combination of vesicles among which there are some with the molecule recognized by the specific antibody employed for quantitation. When the sample is diluted, the number of vesicles covering each bead will be smaller and thus the signal intensity. However, in contrast with other bulk methods, flow cytometry enables the detection of small amounts of particles, because it accumulates the signal of several EVs binding to a bead, also enabling amplification by primary-secondary antibodies. It also has a very good linear response and high sensitivity so it can detect changes in concentration precisely. These advantages make the proposed flow cytometry-based method a superior alternative for the accurate EVs quantitation compared to most commonly used methods.

This quantification method depends on the specific pattern of expression of surface molecules found in the EVs of a given cell type. Therefore, a previous characterization of the EV profile of interest using the same experimental approach is needed. Here we have combined classical EV markers, such as tetraspanins, with other highly abundant membrane proteins, such as the GPI-linked regulatory protein of the complement cascade CD59, which is highly expressed in many cell types. For T-lymphoblasts, MHC-I has also proven to be a very abundant EV marker. Quantitative analysis should be rather performed using those markers with the highest expression levels, so that concentration changes are precisely reflected in changes in MFI and sensitivity of the method is increased. Dim fluorescence intensities involve small signal-to-noise ratios, which prevent the detection of relatively small variations in MFI values. In any case, because not only changes in vesicle number, but also in composition may occur, the ideal scenario will include the parallel assessment of a set of markers for each sample. If EV properties remain unaltered among different samples, quantitation of an unknown sample is very consistent even using different standard curves based on different markers. This implies that changes in EV composition will be also easily detected if within a panel of given markers there is a discrepancy in the behaviour of any of them.

It is known, that a single cell can produce vesicles with different protein content. Some emerging techniques such as immunoaffinity isolation of EVs, may be able to discriminate among different EV subpopulations, but detection limits could be poorer. Moreover, by isolating EV based on single expression marker, all the characterization of those EVs will be skewed from the beginning, being exclusively restricted to those EVs that can be bound to the antibody linked to the beads. On the contrary, aldehyde-sulfate latex beads are densely covered with high affinity binding groups. The combination of unbiased high affinity binding to the beads, with specific and bright antibody detection, rendered a capacity of detection in the range of ng of protein. Conversion of these results to vesicle numbers (based on NTA analyses) suggests that we could detect 3–12 × 10^7^ vesicles, corresponding to a range from 5 × 10^2^ to 3.5 × 10^3^ EVs/bead in the experimental conditions used. These numbers suggest that the upper limit is governed by steric hindrance, so that possibly it could be overcome by using larger beads, as increasing the surface available for vesicle binding would result in higher concentration of vesicles needed to reach the saturation phase of the signal. This should be critical to expand the linear range of detection.

When compared on the same samples to the current “gold standard” in the field (NTA), the bead-based assay showed high accuracy at the lower limit range. NTA is able to give accurate results at lower concentration of vesicles (up to around 3.0 × 10^10^). However, at high concentrations linearity is lost. MFI absolute numbers, in contrast, provide a clear indication of saturation of the measure. In addition, the volume of sample required is much less than for NTA, and acquisition time is shorter. Moreover, NTA cannot discriminate the nature of the particles analysed, thus being unable to distinguish EVs from protein aggregates. Since our method is based on the detection of membrane-bound markers, this drawback is also resolved.

To improve the inter-laboratory robustness of our method, we have analysed some parameters that could affect the outcome of the measurement. In this sense, we have observed that a small amount of protein (0.1% BSA was our routine incubation condition) improves detection. Importantly, performing the coupling in the presence of protein greatly reduced the adherence of EVs to some plastic tubes. Glass vials or low-protein binding plastic tubes are better suited for the coupling of EVs to beads, since they do not compete with latex beads for EV binding.

Finally, although the same methodology could also be used to characterise the composition of EV samples enriched by ultracentrifugation, our results showed that the linear response related to concentration observed with SEC samples is lost with UC samples. This effect could be probably explained by the excess of contaminant proteins in UC samples that affect coupling of EVs to the beads, thus rendering lower MFI values that ultimately hampered linearity.

In summary, here we report a simple and feasible bead-based method for the semi-quantitative characterization of EVs from homogeneous and heterogeneous samples with low protein contamination. This method could be easily adapted to most laboratories with access to basic flow cytometers, thus allowing inter-laboratory comparison and standardization of their results.
